# Virulence Gene Profile and Antimicrobial Susceptibility of *Staphylococcus aureus* Clinical Isolates from the United Arab Emirates

**DOI:** 10.3390/microorganisms14081613

**Published:** 2026-07-24

**Authors:** Wejdan Alamodi, Sara Ali, Marwan Ismail, Praveen Kumar, Salma Elnour Rahma Mohamed

**Affiliations:** Department of Medical Laboratory Sciences, College of Health Sciences, Gulf Medical University, Ajman P.O. Box 4184, United Arab Emiratesdr.sara@gmu.ac.ae (S.A.);

**Keywords:** *Staphylococcus aureus*, MRSA, virulence genes, *mecA*, *PVL*, UAE, antimicrobial resistance, molecular epidemiology, antimicrobial stewardship

## Abstract

*Staphylococcus aureus* causes infections ranging from skin and soft tissue infections to life-threatening systemic disease. The convergence of virulence determinants with antimicrobial resistance, particularly in methicillin-resistant *S. aureus* (MRSA), complicates management. In the United Arab Emirates (UAE), *S. aureus* is a major contributor to community- and hospital-acquired infections, and regional surveillance from the Gulf Cooperation Council has documented a shift toward community-associated MRSA lineages. Despite this clinical burden, granular molecular epidemiological data from the UAE remain scarce. Fifty consecutive *S. aureus* clinical isolates were obtained from Thumbay Laboratories, Ajman, UAE (October 2022–August 2023). Identification and antimicrobial susceptibility testing against 23 agents were performed on the MicroScan WalkAway system. Five virulence genes, namely *mecA*, Panton-Valentine leukocidin (*PVL*; *lukF-S*), exfoliative toxins A (*eta*) and B (*etb*), and toxic shock syndrome toxin (*tst*), were detected by conventional PCR. *PVL* was the most prevalent virulence gene (48%, 24/50), detected exclusively in pus isolates. *mecA* carriage, confirming MRSA, was identified in 44% (22/50), with *PVL*–*mecA* co-occurrence in 22% (11/50). *eta* was present in 6% (3/50), and *etb* and *tst* each in 2% (1/50). Resistance was highest for ampicillin and penicillin (86% each), while full susceptibility (100%) was retained for vancomycin, linezolid, daptomycin, teicoplanin, rifampin, Synercid, and trimethoprim. *mecA* carriage was significantly associated with resistance to amoxicillin, clavulanate, ampicillin, imipenem, penicillin, oxacillin, azithromycin, erythromycin, and ciprofloxacin (*p* < 0.05). This study provides the first integrated molecular characterization of key *S. aureus* virulence determinants, along with a comprehensive antimicrobial susceptibility profile in the UAE. The high *PVL* prevalence and substantial MRSA burden highlight the need for structured regional surveillance and antimicrobial stewardship, while retained susceptibility to last-resort agents offer actionable guidance for empirical therapy.

## 1. Introduction

*Staphylococcus aureus* is a Gram-positive commensal of human skin and mucous membranes that becomes clinically relevant when it breaches host defenses, causing infections ranging from superficial cutaneous lesions to severe systemic disease such as sepsis and endocarditis [1]. Its pathogenicity is driven by a versatile arsenal of virulence determinants (surface adhesins, secreted exotoxins, enzymes, and cell wall components) that mediate tissue adherence, immune evasion, and host–cell damage [2].

Among the secreted toxins of *S. aureus*, Panton-Valentine leukocidin (*PVL*), encoded by *lukF-S*, is a pore-forming cytolytic toxin that targets neutrophils and monocytes, attenuating host immunity and contributing to severe skin and soft tissue infections (SSTIs) and necrotizing pneumonia [3,4]. The exfoliative toxins A and B, encoded by *eta* and *etb*, are serine proteases that cleave desmoglein-1, the principal mediator of epidermal cell adhesion, producing the cutaneous separation characteristic of staphylococcal scalded skin syndrome (SSSS) and bullous impetigo [5,6]. The *tst* gene encodes toxic shock syndrome toxin-1 (TSST-1). This superantigen simultaneously engages major histocompatibility complex class II (MHC-II) and T-cell receptors, driving unrestrained pro-inflammatory cytokine release and the clinical phenotype of toxic shock syndrome (TSS): fever, rash, hypotension, multi-organ failure, and skin desquamation [7].

The emergence of MRSA, driven by the *mecA* gene (which encodes a modified penicillin-binding protein (PBP2a) with reduced β-lactam affinity), has rendered these infections particularly difficult to treat. In 2022, antimicrobial resistance (AMR) was associated with more than one million deaths globally, of which MRSA accounted for a substantial fraction [8]. The interplay between resistance and virulence is especially concerning in MRSA: resistance determinants and virulence factors frequently co-localize on mobile genetic elements and act in concert, amplifying the clinical threat posed by these strains [9].

Within the Gulf Cooperation Council (GCC) region, *S. aureus* and MRSA constitute a substantial share of the clinical bacteriology burden, but the published evidence base is uneven across countries and reporting periods. Hospital surveillance studies from Saudi Arabia, Kuwait, Oman, and the UAE have repeatedly documented MRSA prevalence rates among *S. aureus* isolates exceeding 30% [10,11,12], with healthcare-associated lineages predominating in tertiary centers and community-associated strains increasingly recovered from outpatient skin and soft tissue infections [13]. The GCC also represents a distinctive epidemiological setting: a large expatriate workforce drawn from South Asia, the broader Middle East, North Africa, and sub-Saharan Africa creates continuous opportunities for the importation and circulation of lineages from regions with divergent virulence-gene repertoires and resistance profiles. Despite this exposure, region-specific data on the molecular characteristics of circulating *S. aureus*, particularly the co-occurrence of *mecA* with virulence determinants such as *lukF-S*, *eta*, *etb*, and *tst*, remain sparse, and few UAE-based studies have combined phenotypic resistance characterisation with targeted virulence-gene detection on a defined isolate panel. Regional differences in the distribution of virulence determinants and resistance profiles in *S. aureus* remain incompletely defined, and granular epidemiological data from the UAE are particularly limited. Resolving these patterns at a regional level is essential for refining empirical therapy and for situating local strains within the global epidemiology of *S. aureus*. Accordingly, this study aimed to: (1) characterise confirmed *S. aureus* clinical isolates received from Thumbay Laboratories, including re-confirmation of species identity; (2) determine methicillin resistance phenotypically using the MicroScan WalkAway system; and (3) detect the *mecA* gene together with the virulence genes *eta*, *etb*, *lukF-S*, and *tst* by PCR across the isolate panel.

## 2. Materials and Methods

A descriptive cross-sectional study was conducted from October 2022 to August 2023 at Gulf Medical University, Ajman, UAE, using confirmed *Staphylococcus aureus* clinical isolates provided by Thumbay Laboratories, Ajman, UAE. Fifty consecutive, non-duplicate *S. aureus* isolates were included as a convenience sample. Isolates were recovered from various clinical specimens, including pus, conjunctival swabs, ear swabs, abscesses, body fluids, nasopharyngeal swabs, and skin specimens collected from affiliated hospitals and clinics.

### 2.1. Identification and Antimicrobial Susceptibility Testing

Isolates were received from Thumbay Laboratories as confirmed *S. aureus*, having been identified there using routine clinical microbiology workflows. On receipt at Gulf Medical University, each isolate was sub-cultured on blood agar (HiMedia Laboratories Pvt. Ltd., Mumbai, India) at 37 °C for 24 h, and species identity was re-confirmed in-house. Antimicrobial susceptibility testing against a panel of 23 agents was performed on the MicroScan WalkAway automated system (Beckman Coulter, Brea, CA, USA), generating a complete susceptibility profile for each isolate. Susceptibility results were interpreted according to Clinical and Laboratory Standards Institute (CLSI) breakpoints, document M100, 32nd edition (2022) [14].

### 2.2. Sample Handling and Storage

Isolates were handled under strict aseptic conditions throughout. Each confirmed isolate was re-subcultured in peptone water (HiMedia Laboratories Pvt. Ltd., Mumbai, India) to preserve viability during storage and to support downstream DNA extraction.

### 2.3. DNA Extraction

Genomic DNA was extracted from re-subcultured isolates using the QIAamp DNA Micro Kit (QIAGEN, Hilden, Germany) according to the manufacturer’s protocol. Bacterial pellets were collected by centrifugation at 7500 rpm for 10 min, resuspended in 180 µL lysozyme solution (20 mg/mL; Thermo Fisher Scientific, Waltham, MA, USA), and incubated at 37 °C for at least 30 min. Proteinase K (20 µL; Thermo Fisher Scientific, Waltham, MA, USA) and Buffer AL (200 µL) were added; samples were vortexed and incubated sequentially at 56 °C for 30 min and 95 °C for 15 min. After the addition of 200 µL ethanol (96–100%) and brief pulse-vortexing, lysates were transferred to QIAamp Mini spin columns in 2 mL collection tubes and centrifuged at 8000 rpm for 1 min. Two sequential washes were performed: 500 µL Buffer AW1 (8000 rpm, 1 min) and 500 µL Buffer AW2 (14,000 rpm, 3 min). DNA was eluted with 200 µL Buffer AE after 1 min at room temperature, centrifuged at 8000 rpm for 1 min, and stored at −20 °C until use.

### 2.4. Primer Sequences

Primer pairs were adopted from previously published studies and optimized in-house, with annealing temperatures empirically determined before amplification. Primer sequences, expected amplicon sizes, annealing temperatures, and source references are summarized in Table 1.

### 2.5. PCR Amplification Protocol

PCR screened all 50 *S. aureus* isolates for *mecA*, *eta*, *etb*, *PVL* (*lukF-S*), and *tst*. These five targets were prioritised on the basis of their clinical relevance to *S. aureus* infections reported across the UAE and the wider Gulf Cooperation Council region, their well-characterised associations with SSTIs (*PVL*, *eta*, *etb*) and systemic disease (*mecA*-mediated MRSA, *tst*-mediated toxic shock), as well as the availability of validated, previously published primer sets suitable for conventional PCR. Each 25 µL reaction comprised 12.5 µL of 2× PCR master mix (Thermo Fisher Scientific, Waltham, MA, USA), 1 µL of each forward and reverse primer (Thermo Fisher Scientific, Waltham, MA, USA), 8.5 µL of nuclease-free water, and 2 µL of template DNA. Amplification was performed on a T100 96-Well Thermal Cycler (Bio-Rad Laboratories, Hercules, CA, USA) over 35 cycles. For *mecA* and *tst*, cycling conditions were initial denaturation 94 °C/5 min; denaturation 94 °C/1 min; annealing 56 °C/1 min; extension 72 °C/1 min; final extension 72 °C/7 min; hold at 4 °C. For *eta*, *etb*, and *PVL*, the same general profile was applied with an initial denaturation of 94 °C/3 min, an extension step at 68 °C/1 min, and gene-specific annealing temperatures listed in Table 1. To safeguard assay specificity, every PCR run included a no-template control (nuclease-free water in place of DNA) to monitor for reagent or environmental contamination, together with a positive control consisting of genomic DNA from a previously characterised, gene-positive reference isolate maintained in the laboratory collection.

### 2.6. Gel Electrophoresis

PCR products were resolved on 1.5% (*w*/*v*) agarose gels (Thermo Fisher Scientific, Waltham, MA, USA) prepared in 1× electrophoresis buffer (20 mL of 10× buffer diluted in 980 mL of distilled water), with 1 µL of SYBR Safe DNA gel stain (Thermo Fisher Scientific, Waltham, MA, USA) incorporated into the molten agarose. Gels were allowed to solidify at room temperature for 30 min. Samples (5 µL) were mixed with 2 µL of loading dye (Thermo Fisher Scientific, Waltham, MA, USA) on paraffin wax and loaded alongside a 100 bp DNA ladder (5 µL; Thermo Fisher Scientific, Waltham, MA, USA) as a molecular size reference. Electrophoresis was performed at 100 V for 1 h on a Sub-Cell GT Horizontal Electrophoresis System (Bio-Rad Laboratories, Hercules, CA, USA), and bands were visualized under UV transillumination using a GelDoc Go Imaging System (Bio-Rad Laboratories, Hercules, CA, USA).

### 2.7. Statistical Analysis

Data were entered and analyzed in IBM SPSS Statistics version 30 (IBM Corp., Armonk, NY, USA). Categorical variables were summarized as frequencies and percentages. Associations between virulence gene carriage and specimen type, age group, or gender were assessed using Fisher’s exact test, with statistical significance defined as *p* < 0.05.

## 3. Results

### 3.1. Demographic and Specimen Characteristics of Isolates

A total of 50 *Staphylococcus aureus* clinical isolates were analyzed. Pus specimens accounted for most isolates (Table 2), and most isolates were recovered from male patients and individuals aged 25–35 years.

### 3.2. Virulence Gene Prevalence

Of the 50 isolates, *PVL* (*lukF-S*) was the most prevalent virulence gene (48%, *n* = 24), followed by *mecA* (44%, *n* = 22), *eta* (6%, *n* = 3), and *etb* and *tst* (2% each, *n* = 1). *PVL* and *mecA* co-occurred in 22% of isolates (*n* = 11), whereas no co-occurrence of *eta*, *etb*, or *tst* with *mecA* was observed. Full prevalence data are presented in Table 3.

Stratifying virulence-gene carriage by methicillin susceptibility revealed contrasting profiles between the MRSA (*mecA*-positive, *n* = 22) and MSSA (*mecA*-negative, *n* = 28) subpopulations. *PVL* carriage was identified in 11 of 22 MRSA isolates (50.0%) and in 13 of 28 MSSA isolates (46.4%), indicating that *PVL* was distributed broadly across both subgroups rather than confined to either lineage. The exfoliative toxin genes *eta* (*n* = 3) and *etb* (*n* = 1) were detected exclusively among MSSA isolates, and the single *tst*-positive isolate was likewise MSSA. By contrast, no MRSA isolate carried *eta*, *etb*, or *tst*, such that the only virulence gene observed in the MRSA subpopulation was *PVL*. Considered together with the predominance of pus specimens among *PVL*- and *mecA*-positive isolates (Section 3.1; Supplementary Table S1), these findings indicate that, within this collection, *PVL*-positive MRSA was concentrated in purulent SSTI presentations, while toxin-mediated phenotypes (*eta*, *etb*, *tst*) were observed only on a methicillin-susceptible background.

Per-isolate data for all 50 *S. aureus* isolates, including specimen type, source, virulence gene profile (*mecA*, *eta*, *etb*, *PVL*, *tst*), and full resistance phenotype, are provided in Supplementary Table S1.

### 3.3. Antimicrobial Susceptibility Profile

**Figure 1:**
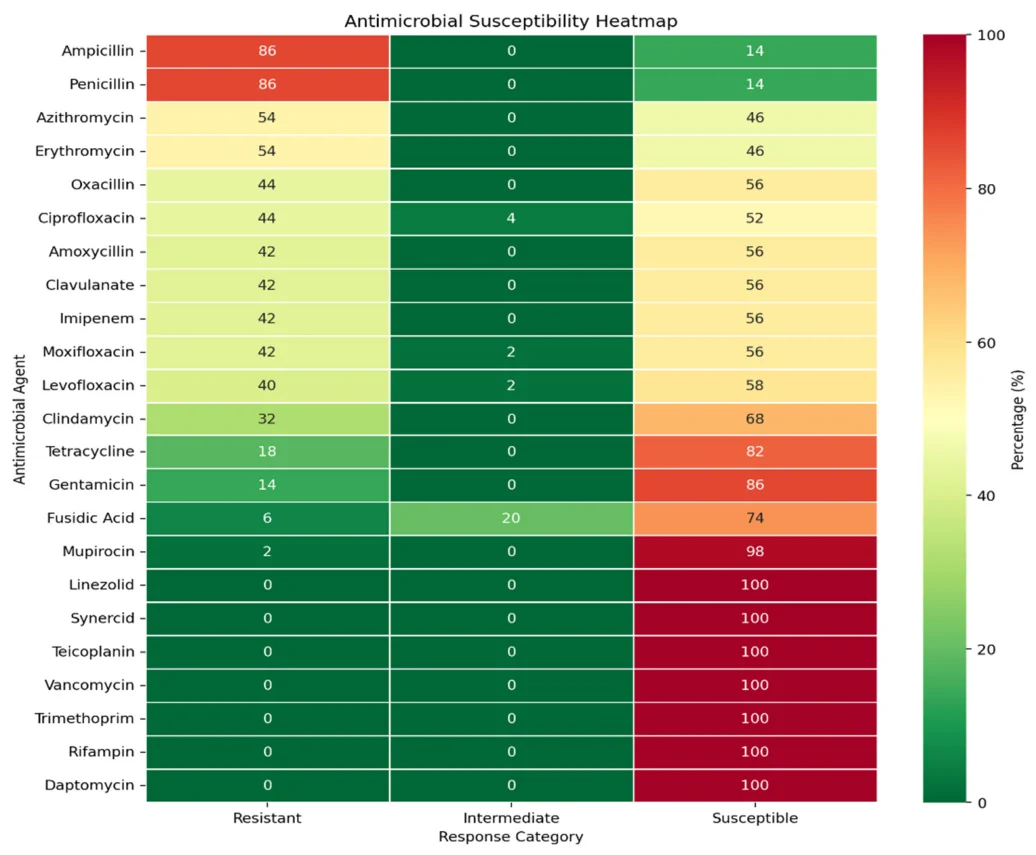
Antimicrobial susceptibility profile of *S. aureus* clinical isolates against 23 agents (*n* = 50). Green shading indicates agents retaining 100% susceptibility across all isolates. Susceptibility interpreted per Clinical and Laboratory Standards Institute (CLSI) breakpoints using the MicroScan WalkAway automated system (Beckman Coulter, Brea, CA, USA).

Antimicrobial susceptibility profiles across 23 agents are presented in Figure 1. Resistance was highest for ampicillin and penicillin (86% each), followed by azithromycin and erythromycin (54% each), ciprofloxacin and oxacillin (44% each), and amoxicillin, clavulanate, imipenem, and moxifloxacin (42% each). Resistance rates for clindamycin, tetracycline, gentamicin, and fusidic acid were 32%, 18%, 14%, and 6%, respectively, with fusidic acid also showing an intermediate rate of 20%. Mupirocin resistance was 2%. Complete susceptibility (100%) was retained for seven agents: vancomycin, linezolid, daptomycin, teicoplanin, rifampin, Synercid, and trimethoprim. Across the full 23-agent panel, overall resistance, intermediate, and susceptibility rates were 28.17%, 1.21%, and 70.34%, respectively.

### 3.4. Distribution of Virulence Genes

The *mecA* gene was predominantly detected in isolates recovered from pus specimens, although no significant associations were observed between *mecA* carriage and specimen type, age group, or gender. In contrast, all *PVL*-positive isolates were recovered exclusively from pus specimens, and *PVL* carriage showed a statistically significant association with specimen type (*p* = 0.036). No significant associations were identified between *PVL* carriage and age or gender. The *eta*, *etb*, and *tst* genes were detected only sporadically among the isolates, with no statistically significant associations observed between their distribution and demographic or specimen-related variables.

### 3.5. Association Between Virulence Gene Carriage and Antimicrobial Resistance

Among *mecA*-positive (MRSA) isolates, resistance was substantially elevated across multiple drug classes: clavulanate, amoxicillin, and imipenem each 95.5%; azithromycin and erythromycin each 77.3%; ciprofloxacin and moxifloxacin each 68.2%; levofloxacin 59.1%; and clindamycin 50%. *mecA* carriage was significantly associated with resistance to amoxicillin, clavulanate, ampicillin, imipenem, penicillin, oxacillin, azithromycin, erythromycin, and ciprofloxacin (*p* < 0.05). In contrast, no statistically significant association was identified between *PVL*, *eta*, *etb*, or *tst* carriage and any of the tested antimicrobials.

## 4. Discussion

To our knowledge, this is the first study from the UAE to integrate molecular characterization of *mecA*, *eta*, *etb*, *PVL*, and *tst* with full 23-agent antimicrobial susceptibility profiling in *S. aureus* clinical isolates. The findings situate local strains within regional and global molecular epidemiology. The *mecA*-confirmed MRSA prevalence of 44% sits within the moderate-to-high range reported globally [8], lower than rates from Iranian hospital and ICU cohorts (86.3–93.3%) [18,19] but higher than surgical-site isolates from Bangladesh (21%) [20]. The balanced distribution between genders argues against gender-restricted transmission and supports broadly applied infection-control measures. The strong predominance of *mecA* in pus specimens (77.3%) is concordant with previous reports [18,19,21] and reinforces the established link between MRSA and skin and soft tissue infections (SSTIs); the absence of *mecA* in our few blood isolates is most plausibly a sampling artefact given limited bloodstream sampling.

The most clinically salient finding is the 48% prevalence of *PVL*, detected exclusively in pus. This sits at the upper end of the GCC range (35–45%) [10] and aligns with CA-MRSA cohorts from Saudi Arabia (49%) [13], China (45.1%) [22], and Bangladesh (42.8%) [20]. It substantially exceeds reported Iranian rates (5.45–21.4%) [19,23,24]. The exclusive detection in pus is consistent with the established role of *PVL* in suppurative SSTIs [3,4]. Together with the regional shift toward CA-MRSA dominance documented in UAE and GCC surveillance [10,11,12], this is consistent with, though cannot confirm, a molecular profile similar to globally disseminated CA-MRSA clones. The *PVL*–specimen-type association was statistically significant (*p* = 0.036); no associations with age or gender were observed, indicating that *PVL*-associated infections span a broad demographic.

*eta* was detected in 6% of isolates (two pus, one conjunctival swab), and *etb* in 2% (a single conjunctival swab). The *eta* rate aligns with reports from Iran (0.68%) [23] and Iraq (2.63%) [21], but contrasts sharply with 94.4% reported by Koosha et al. [18]. *etb* is consistent with the 1.3–3.1% range reported in most comparable cohorts [21,23,24]. *tst* was detected in 2% (a single pus specimen), comparable to Potindji et al. (2.63%) [21] but markedly lower than Iranian ICU cohorts (51.4%) [19,24] and Indian data (50.79%) [25]. These differences likely reflect the predominantly outpatient, SSTI-associated character of our cohort relative to the ICU- and hospitalized populations in those studies. The single ocular *eta*-positive isolate is hypothesis-generating and warrants confirmation in larger cohorts.

The antimicrobial susceptibility profile reveals several clinically important patterns. Ampicillin and penicillin resistance of 86% is consistent with global trends [18,19,24]. MRSA isolates showed a multidrug-resistant phenotype, with high resistance to macrolides, fluoroquinolones, and β-lactam/carbapenem combinations. This pattern is consistent with co-carriage of resistance determinants on mobile genetic elements in *mecA*-positive strains. Critically, daptomycin, linezolid, vancomycin, trimethoprim, teicoplanin, Synercid, and rifampin retained 100% susceptibility, confirming the continued utility of these agents in the UAE context [18,19,22,24]. Ciprofloxacin resistance was 44% overall and 68.2% among MRSA isolates, lower than rates from Iran (71.4%) [24] and Iraq (97.72%) [26], but a clear concern for empirical regimens.

These findings carry direct implications for UAE practice. The convergence of 44% MRSA prevalence with 48% *PVL* carriage argues for empirical anti-MRSA coverage in moderate-to-severe SSTIs. Toxin-suppressing agents (clindamycin where D-test-negative, or linezolid) should be considered as adjuncts in necrotizing presentations, consistent with IDSA guidance [27]. The retained 100% susceptibility to the last-line agents above must be protected through formal stewardship, particularly given erythromycin/azithromycin resistance approaching 54%. The molecular profile is consistent with the broader GCC shift toward CA-MRSA lineages [10,11,12] and supports prospective multicenter molecular surveillance using SCCmec and *spa* typing or whole-genome sequencing.

Placing the present findings as a snapshot within longitudinal UAE-specific data adds further context. Thomsen and colleagues’ 12-year retrospective analysis of *S. aureus* bloodstream isolates from a tertiary UAE center documented a progressive increase in methicillin resistance alongside sustained glycopeptide susceptibility and a rising contribution of community-associated lineages [10,11]. The 44% *mecA* carriage rate in our outpatient- and SSTI-skewed cohort is broadly consistent with the upper end of that trajectory, and the exclusive detection of *PVL* in pus specimens accords with the SSTI-predominant CA-MRSA pattern described regionally [10,11,12]. Differences in specimen origin and care setting between the bloodstream-focused Thomsen dataset and the present SSTI-predominant collection warrant cautious comparison, but together they reinforce a coherent picture of CA-MRSA-compatible profiles, high *PVL* carriage on a methicillin-resistant background with retained last-line susceptibility, as an increasingly stable feature of the UAE *S. aureus* landscape.

From a clinical standpoint, the convergence of high *PVL* prevalence and high *mecA* carriage has practical implications for empirical management of purulent SSTIs in UAE adult and paediatric outpatient settings, where a low threshold for incision-and-drainage, microbiological sampling, and *PVL*-aware antibiotic selection (including toxin-suppressing adjuncts in necrotising or recurrent presentations) is warranted. The 100% susceptibility retained by vancomycin, linezolid, daptomycin, teicoplanin, trimethoprim, quinupristin/dalfopristin (Synercid), and rifampin defines a defensible last-line formulary, but the 54% macrolide resistance and 44% ciprofloxacin resistance markedly narrow the empirical oral options and argue for facility-level antibiograms to guide first-line prescribing. We emphasise, however, that the absence of granular clinical metadata, admission status, prior antimicrobial exposure, comorbidities, treatment trajectories, and patient outcomes means that these implications are derived from the molecular and phenotypic profile of the isolates rather than from observed clinical correlations, and should be regarded as hypothesis-generating rather than evidence of differential clinical performance.

Set against the broader regional literature, the gene-frequency profile in this Sharjah-based cohort occupies an intermediate position informative for UAE-specific antimicrobial stewardship. The 44% *mecA* carriage rate exceeds figures historically cited for community-onset *S. aureus* across several GCC settings yet aligns with the upward MRSA trajectory documented for the UAE over the last decade [10,11]. *PVL*-positive isolates were recovered across both MRSA and MSSA backgrounds, with toxin-gene carriage concentrated in pus specimens, empirical therapy for purulent SSTIs in the UAE should therefore not assume methicillin susceptibility on the basis of community acquisition alone, and the exclusive detection of *eta*, *etb*, and *tst* within the MSSA subset cautions against treating MSSA isolates as intrinsically low-risk. These observations argue for incorporating routine molecular surveillance, even at the level of a focused gene panel, into UAE clinical-microbiology workflows to inform locally calibrated empirical-therapy guidance.

Several limitations should be acknowledged. The single-center design and sample size of 50 isolates constrain generalizability, and the cross-sectional design precludes temporal-trend analysis. PCR-based detection establishes gene presence but not transcriptional activity or functional toxin production. The absence of whole-genome sequencing precluded clonal-lineage assignment, and incomplete clinical metadata for some isolates limited epidemiological inference. The predominance of pus specimens (84%) may have inflated the apparent *PVL* prevalence given *PVL*’s established tropism for suppurative SSTIs; findings should therefore be interpreted with caution in non-SSTI clinical contexts. The unavailability of specific clinical metadata, including hospitalization history, prior antimicrobial therapy, comorbidities, infection severity, and clinical outcomes, precluded the correlation of molecular findings with clinical presentation. Given the cohort size (*n* = 50) and the resulting low expected cell frequencies in cross-tabulations of virulence-gene carriage against specimen type, age group, and gender, Fisher’s exact test was used in preference to the chi-squared approximation as the appropriate inferential test for sparse categorical data. Multivariate analyses adjusting simultaneously for specimen type, demographic factors, and co-carriage patterns were not attempted, as the cohort is underpowered to support reliable estimation of adjusted effects across multiple covariates; reported associations should accordingly be interpreted as unadjusted and hypothesis-generating. Multicenter, longitudinal studies incorporating whole-genome sequencing are needed to address these limitations.

## 5. Conclusions

In 50 *S. aureus* clinical isolates from the UAE, *PVL* (48%, exclusively in pus) and *mecA*-confirmed MRSA (44%) were the dominant virulence and resistance markers, with *eta*, *etb*, and *tst* detected only sporadically. Despite high ampicillin and penicillin resistance (86%), full susceptibility was retained to vancomycin, linezolid, daptomycin, teicoplanin, trimethoprim, Synercid, and rifampin, supporting their continued role as last-resort options.

These findings underscore the need for (1) sustained, structured surveillance of *S. aureus* virulence-gene distribution across UAE healthcare facilities; (2) empirical treatment strategies informed by local resistance and virulence data; (3) robust antimicrobial stewardship programs to safeguard the efficacy of last-resort agents; and (4) multicenter studies incorporating whole-genome sequencing to comprehensively define the molecular epidemiology of *S. aureus* across the UAE and the broader Middle East and North Africa (MENA) region.

Beyond the five targets prioritised here on the basis of regional clinical relevance and established SSTI/systemic-disease associations, future UAE-based work should extend the molecular profile to include SCCmec typing and *agr* typing to resolve the lineage architecture underlying the CA-MRSA-compatible patterns observed in this cohort, together with screening for additional enterotoxin (*sea–see*) and adhesin gene families that were beyond the scope of the present panel. Coupling such expanded molecular characterisation with a structured collection of clinical metadata, including admission status, prior antimicrobial exposure, comorbidities, and clinical outcomes, would allow the molecular signatures described here to be linked directly to disease severity, treatment response, and transmission dynamics across UAE healthcare settings.

## Figures and Tables

**Table 1:** Primer sequences, annealing temperatures, and expected amplicon sizes for virulence gene PCR detection.

Target Gene	Primer Sequence (5′→3′) F/R	Product Size (bp)	Annealing Temp (°C)	Reference
*mecA* (methicillin resistance)	GTAGAAATGACTGAACGTCCGATAA CCAATTCCACATTGTTTCGGTCTAA	310	56	[15]
*eta* (exfoliative toxin A)	ACTGTAGGAGCTAGTGCATTTGT TGGATACTTTTGTCTATCTTTTTCATCAAC	190	57	[16]
*etb* (exfoliative toxin B)	CAGATAAAGAGCTTTATACACACATTAC AGTGAACTTATCTTTCTATTGAAAAACACTA	612	57	[16]
*PVL* (*lukF-S*)	ATCATTAGGTAAAATGTCTGGACATGATCCA GCATCAAGTGTATTGGATAGCAAAAGC	433	50	[17]
*tst* (toxic shock syndrome toxin)	TTCACTATTTGTAAAAGTGTCAGACCCACT TACTAATGAATTTTTTTATCGTAAGCCCTT	180	56	[15]

**Table 2:** Distribution of *S. aureus* clinical isolates by specimen type (*n* = 50).

Specimen Type	Frequency (*n*)	Percentage (%)
Pus	42	84
Conjunctival swab	2	4
Ear swab	2	4
Abscess	1	2
Body fluid	1	2
Nasopharyngeal swab	1	2
Skin	1	2
Total	50	100

**Table 3:** Prevalence of virulence genes in *S. aureus* clinical isolates (*n* = 50).

Virulence *Gene*	No. Positive Isolates (*n*)	Prevalence (%)
*PVL* (*lukF-S*)	24	48
*mecA*	22	44
*eta*	3	6
*etb*	1	2
*tst*	1	2
*mecA* + *PVL* (co-occurrence)	11	22

## Data Availability

The original contributions presented in this study are included in the article. Further inquiries can be directed to the corresponding author.

## Supplementary Materials

The following supporting information can be downloaded at: https://www.mdpi.com/article/10.3390/microorganisms14081613/s1, Table S1: Virulence Gene Profiles and Antimicrobial Resistance Patterns of *Staphylococcus aureus* Clinical Isolates.
