# Supplementary material for: Virulence Gene Profile and Antimicrobial Susceptibility of Staphylococcus aureus Clinical Isolates from the United Arab Emirates
Source: Microorganisms. 2026 Jul 24;14(8):1613. doi: 10.3390/microorganisms14081613 (PMC13515636; doi:10.3390/microorganisms14081613)
Supplement: Supplementary file 1 [file microorganisms-14-01613-s001.zip › microorganisms-4333143-supplementary.pdf]

Table S1. Virulence Gene Profiles and Antimicrobial Resistance Patterns of *Staphylococcus aureus* Clinical Isolates.

Isolate Information			Clinical Source		Virulence Gene Profile					Antimicrobial Resistance Profile (R/I/S — CLSI M100, 32nd ed. 2022)									
**Isolate No.**	**Sample ID**	**Collection Year**	**Specimen Type**	**Source Facility**	**mecA**	**eta**	**etb**	**PVL**	**tst**	**OXA**	**VAN**	**TEC**	**GEN**	**CIP**	**ERY**	**CLI**	**SXT**	**RIF**	**TET**
**SA-01**	TL-2022-001	2022	Pus	Thumbay Laboratories, Ajman	+	−	−	+	−	**R**	S	S	**R**	**R**	**R**	**R**	S	S	**R**
**SA-02**	TL-2022-002	2022	Pus	Thumbay Laboratories, Ajman	+	−	−	+	−	**R**	S	S	**R**	**R**	**R**	**R**	S	S	**R**
**SA-03**	TL-2022-003	2022	Pus	Thumbay Laboratories, Ajman	+	−	−	+	−	**R**	S	S	**R**	**R**	**R**	**R**	S	S	**R**
**SA-04**	TL-2022-004	2022	Pus	Thumbay Laboratories, Ajman	+	−	−	+	−	**R**	S	S	**R**	**R**	**R**	**R**	S	S	**R**
**SA-05**	TL-2022-005	2022	Pus	Thumbay Laboratories, Ajman	+	−	−	+	−	**R**	S	S	**R**	**R**	**R**	**R**	S	S	**R**
**SA-06**	TL-2022-006	2022	Pus	Thumbay Laboratories, Ajman	+	−	−	+	−	**R**	S	S	**R**	**R**	**R**	**R**	S	S	**R**
**SA-07**	TL-2022-007	2022	Pus	Thumbay Laboratories, Ajman	+	−	−	+	−	**R**	S	S	**R**	**R**	**R**	**R**	S	S	S
**SA-08**	TL-2022-008	2022	Pus	Thumbay Laboratories, Ajman	+	−	−	+	−	**R**	S	S	S	**R**	**R**	**R**	S	S	S
**SA-09**	TL-2022-009	2022	Pus	Thumbay Laboratories, Ajman	+	−	−	+	−	**R**	S	S	S	**R**	**R**	**R**	S	S	S
**SA-10**	TL-2022-010	2022	Pus	Thumbay Laboratories, Ajman	+	−	−	+	−	**R**	S	S	S	**R**	**R**	**R**	S	S	S
**SA-11**	TL-2022-011	2022	Pus	Thumbay Laboratories, Ajman	+	−	−	+	−	**R**	S	S	S	**R**	**R**	**R**	S	S	S
**SA-12**	TL-2022-012	2022	Pus	Thumbay Laboratories, Ajman	+	−	−	+	−	**R**	S	S	S	**R**	**R**	S	S	S	S
**SA-13**	TL-2022-013	2022	Pus	Thumbay Laboratories, Ajman	+	−	−	+	−	**R**	S	S	S	**R**	**R**	S	S	S	S
**SA-14**	TL-2022-014	2022	Pus	Thumbay Laboratories, Ajman	+	−	−	+	−	**R**	S	S	S	**R**	**R**	S	S	S	S
**SA-15**	TL-2022-015	2022	Pus	Thumbay Laboratories, Ajman	+	−	−	+	−	**R**	S	S	S	**R**	**R**	S	S	S	S
**SA-16**	TL-2022-016	2022	Pus	Thumbay Laboratories, Ajman	+	−	−	+	−	**R**	S	S	S	S	**R**	S	S	S	S
**SA-17**	TL-2022-017	2022	Pus	Thumbay Laboratories, Ajman	+	−	−	+	−	**R**	S	S	S	S	**R**	S	S	S	S
**SA-18**	TL-2022-018	2022	Conjunctival swab	Thumbay Laboratories, Ajman	+	−	−	+	−	**R**	S	S	S	S	S	S	S	S	S
**SA-19**	TL-2022-019	2022	Ear swab	Thumbay Laboratories, Ajman	+	−	−	+	−	**R**	S	S	S	S	S	S	S	S	S
**SA-20**	TL-2022-020	2022	Skin	Thumbay Laboratories, Ajman	+	−	−	+	−	**R**	S	S	S	S	S	S	S	S	S
**SA-21**	TL-2022-021	2022	Abscess	Thumbay Laboratories, Ajman	+	−	−	+	−	**R**	S	S	S	S	S	S	S	S	S
**SA-22**	TL-2022-022	2022	Nasopharyngeal swab	Thumbay Laboratories, Ajman	+	−	−	+	−	**R**	S	S	S	S	S	S	S	S	S
**SA-23**	TL-2022-023	2022	Pus	Thumbay Laboratories, Ajman	−	−	−	+	−	S	S	S	S	**R**	**R**	**R**	S	S	**R**
**SA-24**	TL-2022-024	2022	Pus	Thumbay Laboratories, Ajman	−	−	−	+	−	S	S	S	S	**R**	**R**	**R**	S	S	**R**
**SA-25**	TL-2022-025	2022	Pus	Thumbay Laboratories, Ajman	−	+	−	−	−	S	S	S	S	**R**	**R**	**R**	S	S	**R**
**SA-26**	TL-2022-026	2023	Pus	Thumbay Laboratories, Ajman	−	+	−	−	−	S	S	S	S	**R**	**R**	**R**	S	S	S
**SA-27**	TL-2022-027	2023	Conjunctival swab	Thumbay Laboratories, Ajman	−	+	−	−	−	S	S	S	S	**R**	**R**	**R**	S	S	S
**SA-28**	TL-2022-028	2023	Conjunctival swab	Thumbay Laboratories, Ajman	−	−	+	−	−	S	S	S	S	**R**	**R**	S	S	S	S

**SA-29**	TL-2022-029	2023	Pus	Thumbay Laboratories, Ajman	–	–	–	–	+	S	S	S	S	**R**	**R**	S	S	S	S
**SA-30**	TL-2022-030	2023	Pus	Thumbay Laboratories, Ajman	–	–	–	–	–	S	S	S	S	**I**	**R**	S	S	S	S
**SA-31**	TL-2022-031	2023	Pus	Thumbay Laboratories, Ajman	–	–	–	–	–	S	S	S	S	**I**	**R**	S	S	S	S
**SA-32**	TL-2022-032	2023	Pus	Thumbay Laboratories, Ajman	–	–	–	–	–	S	S	S	S	S	**R**	S	S	S	S
**SA-33**	TL-2022-033	2023	Pus	Thumbay Laboratories, Ajman	–	–	–	–	–	S	S	S	S	S	S	S	S	S	S
**SA-34**	TL-2022-034	2023	Pus	Thumbay Laboratories, Ajman	–	–	–	–	–	S	S	S	S	S	S	S	S	S	S
**SA-35**	TL-2022-035	2023	Pus	Thumbay Laboratories, Ajman	–	–	–	–	–	S	S	S	S	S	S	S	S	S	S
**SA-36**	TL-2022-036	2023	Pus	Thumbay Laboratories, Ajman	–	–	–	–	–	S	S	S	S	S	S	S	S	S	S
**SA-37**	TL-2022-037	2023	Pus	Thumbay Laboratories, Ajman	–	–	–	–	–	S	S	S	S	S	S	S	S	S	S
**SA-38**	TL-2022-038	2023	Pus	Thumbay Laboratories, Ajman	–	–	–	–	–	S	S	S	S	S	S	S	S	S	S
**SA-39**	TL-2022-039	2023	Pus	Thumbay Laboratories, Ajman	–	–	–	–	–	S	S	S	S	S	S	S	S	S	S
**SA-40**	TL-2022-040	2023	Pus	Thumbay Laboratories, Ajman	–	–	–	–	–	S	S	S	S	S	S	S	S	S	S
**SA-41**	TL-2022-041	2023	Pus	Thumbay Laboratories, Ajman	–	–	–	–	–	S	S	S	S	S	S	S	S	S	S
**SA-42**	TL-2022-042	2023	Pus	Thumbay Laboratories, Ajman	–	–	–	–	–	S	S	S	S	S	S	S	S	S	S
**SA-43**	TL-2022-043	2023	Pus	Thumbay Laboratories, Ajman	–	–	–	–	–	S	S	S	S	S	S	S	S	S	S
**SA-44**	TL-2022-044	2023	Pus	Thumbay Laboratories, Ajman	–	–	–	–	–	S	S	S	S	S	S	S	S	S	S
**SA-45**	TL-2022-045	2023	Pus	Thumbay Laboratories, Ajman	–	–	–	–	–	S	S	S	S	S	S	S	S	S	S
**SA-46**	TL-2022-046	2023	Pus	Thumbay Laboratories, Ajman	–	–	–	–	–	S	S	S	S	S	S	S	S	S	S
**SA-47**	TL-2022-047	2023	Pus	Thumbay Laboratories, Ajman	–	–	–	–	–	S	S	S	S	S	S	S	S	S	S
**SA-48**	TL-2022-048	2023	Pus	Thumbay Laboratories, Ajman	–	–	–	–	–	S	S	S	S	S	S	S	S	S	S
**SA-49**	TL-2022-049	2023	Pus	Thumbay Laboratories, Ajman	–	–	–	–	–	S	S	S	S	S	S	S	S	S	S
**SA-50**	TL-2022-050	2023	Pus	Thumbay Laboratories, Ajman	–	–	–	–	–	S	S	S	S	S	S	S	S	S	S
